# Overcoming diffusion limits to advance brain organoids

**DOI:** 10.4103/NRR.NRR-D-25-01296

**Published:** 2026-01-27

**Authors:** Xuyu Qian

**Affiliations:** Division of Neurology, Department of Pediatrics, Perelman School of Medicine, University of Pennsylvania, Philadelphia, PA, USA; Center for Brain Research Initiative in Development, Genetics, and Engineering, Children’s Hospital of Philadelphia Research Institute, Philadelphia, PA, USA; Department of Neuroscience, Perelman School of Medicine, University of Pennsylvania, Philadelphia, PA, USA

Human pluripotent stem cell–derived brain organoids have become a central tool for modeling human neurodevelopment. Compared to traditional *in vitro* systems, brain organoids are distinguished by their ability to allow cells to self-organize and follow intrinsic developmental programs, thereby dynamically recapitulating aspects of *in vivo* brain development. Cerebral cortex organoids, for instance, can robustly mimic many features of human brain formation during mid-gestation. However, current methodologies remain largely restricted to modeling processes that occur prior to the late second trimester.

**Figure 1:**
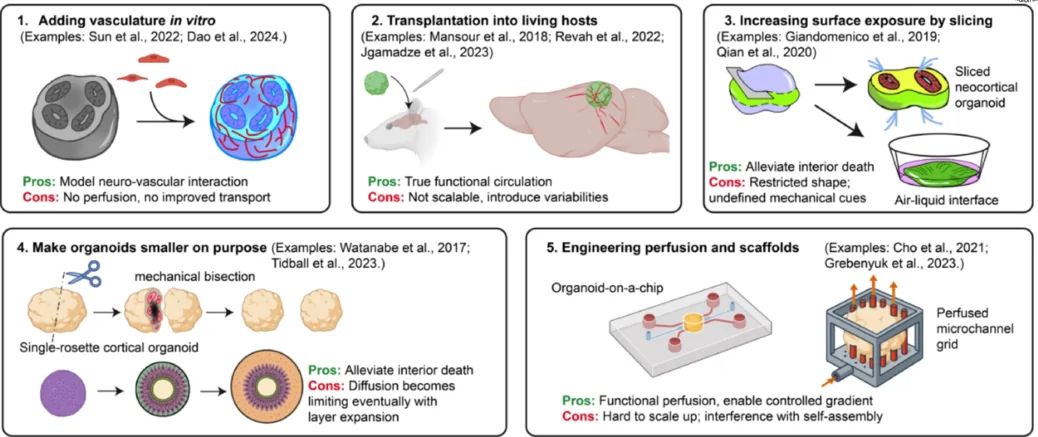
Summary of current approaches to alleviate diffusion limits in organoids. Representative strategies designed to increase oxygen and nutrient delivery are illustrated. Each has delivered meaningful gains and their trade-offs. Part of the illustration was created with BioRender.com.

The primary barrier to advancing organoid models beyond this stage is the development of interior hypoxia accompanied by necrotic cell death. As organoids expand, diffusion alone becomes insufficient to sustain their increasing metabolic demands. The resulting cell death disrupts radial glial organization, destabilizes ventricular structures, and halts the production of later-born cell types. To address this problem, the field has converged on three broad directions: (1) introducing vasculature, either *in vitro* or through transplantation into animals; (2) increasing surface exposure to the culture environment, either by slicing or by constraining organoids to remain small; and (3) applying engineering solutions to deliver controlled flow or create channels within or around the tissue. Each approach has yielded measurable progress, yet none has provided a complete solution for sustaining large, long-lived, and fully self-assembled cortical tissue. This perspective examines the strengths and limitations of each of these strategies and considers how emerging approaches may help overcome the fundamental challenge of sustaining viable, structurally organized, and developmentally advanced brain organoids (**Figure 1**).

**Current approaches: *Adding vasculature in vitro:*** An intuitive answer to address organoid hypoxia is to introduce endothelial cells into brain organoids in defined co-cultures. Several groups have developed “brain-vessel assembloids,” combining brain organoids with vascular organoids or endothelial progenitors to model neurovascular interactions and blood–brain barrier properties in a human context (Sun et al., 2022; Dao et al., 2024). These systems generate endothelial networks with tight junction proteins, pericyte association, and barrier-like transport features. As model systems, they are valuable because endothelial cues influence neural progenitor behavior as well as neuronal and glial maturation. However, these systems do not resolve interior hypoxia. The endothelial networks that form are typically tube-like but discontinuous: many segments terminate in blind ends, lack extensive interconnections, and are not connected to defined inlet-outlet reservoirs. In the absence of a pressure gradient, there is no sustained, pressure-driven flow through the network, so mass transport remains diffusion-limited. Even where short lumen segments contact the medium, their small diameters (typically less than 5 µm) confer very high hydraulic resistance (which scales inversely with the fourth power of radius), making appreciable convective throughput impractical without imposing substantial pressure. Moreover, the medium itself has a very limited oxygen-carrying capacity compared to red blood cells and cannot supply the interior demand. Therefore, the endothelial structures are introduced to provide local cell-cell interaction cues rather than acting as functional conduits that deliver oxygen and nutrients to the organoid core.

***Transplantation into living hosts:*** Transplanting brain organoids into an animal host, typically the rodent brain, is currently the most effective way to sustain organoid survival and maturation, because invading host vessels establish true, pressure-driven perfusion. In rodent hosts, vascular ingrowth occurs within weeks after transplantation, and markers of perfusion, oxygenation, and metabolic health in organoid grafts improve markedly relative to *in vitro* culture (Mansour et al., 2018). Studies have demonstrated that grafted organoid neurons exhibit morphological and functional maturation far beyond what is achievable *in vitro* (Revah et al., 2022; Jgamadze et al., 2023). Transplantation also permits observation of long-range axon growth and target selection. Oligodendrocytes, the cells that form myelin, also mature more completely *in vivo* than *in vitro*, with mature myelination detectable at later time points. Nevertheless, these gains come with practical and conceptual costs. Transplantation is labor-intensive and low-throughput, introducing substantial technical variability and constraining the types of experiments that can be performed. It is straightforward to grow hundreds of organoids simultaneously, but impractical to transplant each into an individual rodent and subsequently euthanize the animals to recover grafts for analysis. Host-graft interactions are difficult to control and interpret. Species mismatch introduces nonhuman cytokine, hormonal, and extracellular-matrix environments that shape lineage progression and synaptic maturation. Microglial and immune contributions differ from human settings. Ethical concerns surrounding these experiments also remain controversial. In short, transplantation demonstrates what is possible when fully functional circulation is achieved, but it does not yet provide a scalable discovery platform while retaining the advantages that make organoids such a favorable model system.

***Increasing surface exposure by slicing:*** Reducing diffusion distance by changing organoid geometry, essentially bringing interior cells closer to the surface, is another effective strategy. By slicing organoids with a vibratome and culturing them at an air–liquid interface, oxygen exposure is maximized. These “air–liquid interface cortical organoids” have more robust long-range tract-like projections compared with free-floating controls (Giandomenico et al., 2019). Sliced neocortical organoids also use vibratome sectioning to reduce diffusion distance while preserving ventricular zones and radial glia scaffolds within the three-dimensional structure of 500-µm-thick sections (Qian et al., 2020). Sliced neocortical organoids are re-sectioned monthly in the same orientation to prevent thickening and to maintain conditions that favor oxygen exchange. In sliced neocortical organoids, production and radial migration of neurons persist for at least 150 days in culture, enabling the emergence of distinct deep and upper cortical layers. These methods have limitations. They are more labor-intensive, particularly if repeated slicing is required. Tissue geometry is altered during sectioning and remains constrained by the slice, and “air–liquid interface cortical organoids” can undergo substantial flattening that compromises the three-dimensional integrity of ventricular structures. It also remains unclear whether the mechanical perturbation associated with slicing imparts signals that alter intrinsic developmental programs. Despite these constraints, slicing remains an effective and practical way to avert interior necrosis while preserving much of the self-organized architecture that makes organoids informative.

***Making organoids smaller on purpose:*** Because interior hypoxia emerges as organoids grow, researchers have explored ways to deliberately limit the size of organoids. A simple method is routine mechanical bisection to keep organoids below diffusion limits: organoids are periodically cut in half with scissors or scalpel, the pieces round up, and culture continues as separate spheroids (Watanabe et al., 2017). The trade-off is obvious: cuts risk disrupting the integrity of self-assembled architectures. A more refined approach is to grow self-organizing, single-rosette cortical organoids (SOSR-COs) (Tidball et al., 2023). In contrast to conventional cortical organoids, which often contain multiple, sometimes dozens, of ventricular structures arising from spontaneous self-assembly, SOSR-COs standardize early morphogenesis around a single ventricular rosette and maintain a small overall diameter. These constructs exhibit reduced interior hypoxia and improved batch-to-batch uniformity. The limitation, however, remains in the dimension: in the study reporting SOSR-Cos (Tidball et al., 2023), apical-to-basal thickness did not exceed 300-400 μm, short of the expanded cortical architectures characterized by an expanded outer subventricular zone and distinct deep and upper neuronal layers. Because late developmental events require both time and tissue scale, miniaturized constructs may not fully capture these features before diffusion becomes limiting again. Nevertheless, the single-rosette paradigm is promising; further optimization of culture conditions should yield cortical organoids that are substantially healthier than traditional counterparts.

***Engineering perfusion and scaffolds:*** Engineering strategies aim to impose controlled convective transport in or around brain organoids so that oxygen and nutrients reach depths that are diffusion-limited in ordinary culture. One approach integrates organoids with microfluidic devices, often called organ-on-a-chip, by placing them on microfluidic devices where channels run beneath or around the tissue. Periodic, low-shear perfusion stabilizes medium composition at the interface, improves survival, and supports thicker cortical-like zones and more mature electrophysiology than static culture, with options to integrate oxygen, pH, and metabolite sensors for long-term, defined conditions (Cho et al., 2021). A second approach embeds microchannels within soft materials that connect to external pumps. These “soft microfluidic” networks reduce inner-cell death and support more advanced maturation while allowing precise control of flow (Grebenyuk et al., 2023). A third approach, demonstrated as proof of concept in non-neural three-dimensional tissues, fabricates patterned conduits or perfusable voids within hydrogels. These branched networks support continuous flow through centimeter-scale constructs and, although validated outside the brain context, the same hydrogel printing and perfusion principles may be adaptable to cerebral organoids (Grigoryan et al., 2019). Across these systems, two recurring challenges remain. First, interference with self-assembly: ventricular expansion and radial glial alignment are sensitive to mechanical constraint, and even compliant lattices impose boundary conditions that can bias cortical morphogenesis. Second, throughput: many devices culture a single organoid with bespoke plumbing, which is suitable for demonstrations but not for discovery-scale experiments that require dozens of conditions and replicates.

**Future directions:** Current work has provided crucial proof of principle. In the short term, further advancing and refining the methodology of controlling organoid size/geometry and scaling up engineering will remain the most effective and attainable approaches. One particularly intriguing avenue extends the size/geometry strategy through the development of neural tube-like organoids, which model early neuroepithelial tube formation under morphogen gradients (Xue et al., 2024). These models provide a defined starting geometry for forebrain trajectories and, importantly, remain small and shape-controlled. The elongated geometry can, in principle, permit laminar structures to extend further than in spherical organoids before diffusion becomes the dominant constraint. At present, demonstrations have been maintained for relatively short culture periods that mimic early first-trimester morphogenesis and have not yet shown a cortical plate outside the neuroepithelium. Further optimization aimed at sustaining neurogenesis and radial migration is required to enable formation of a subventricular zone and cortical plate with extended culture.

In parallel, the engineering that enables perfusion using scaffolds and organ-on-a-chip needs to be scaled for experiments rather than demonstrations. The goal is not a single heroic device, but a manufacturable, plate-format system that supports dozens or even hundreds of organoids at once. Meanwhile, it is important that the device leave self-assembly as unconstrained as possible by avoiding rigid walls and intrusions into the organoid interior. The necessary components already exist: soft, perfusable networks and chip-based flow control, which now need to be combined into routine, arrayed formats that laboratories can run repeatedly.

Finally, vascularization with functional circulation, achieved by integrating engineering with development, remains the hallmark solution to the problem. An engineering path forward will focus on vascularization with microfluidics, inspired by platforms that already achieve functional intraluminal flow in non-neural systems (Quintard et al., 2024). Devices that assemble endothelial networks around mesenchymal and pancreatic islet spheroids, and around blood-vessel organoids, demonstrate anastomosis and continuous perfusion on-chip with improved growth and function over weeks. Adapting this approach to brain organoids will require perfusable networks that interface with neuroepithelium without rigid constraints that distort ventricular geometry or radial scaffolds.

Complementing this, developmentally inspired co-differentiation may offer superior integration. In endodermal organoids, simultaneous specification of epithelial and mesenchymal lineages yields better vasculature than add-on endothelium (Miao et al., 2025). By analogy, brain organoids could co-differentiate mesodermal progenitors that generate pericytes and vascular smooth muscle, and myeloid progenitors that generate microglia, while preserving the morphogenetic program of neuroectoderm. The balance is delicate, but the rationale is clear: co-differentiation provides the right partners at the right time, making it the optimal route to functional vascularization.

**Conclusion:** Brain organoids have already transformed the study of human neurodevelopment, providing access to dynamic processes that traditional systems could not capture. Yet their greatest limitation, interior hypoxia, remains the key barrier to modeling later stages of cortical development. The field has responded with creative solutions that offer important advantages while also underscoring the difficulty of sustaining large, long-lived, structurally faithful tissue *in vitro*. Looking ahead, the path forward will likely be plural. Continued refinement of current methods, coupled with new innovations such as neural tube-like geometries, scaled engineering platforms, and developmentally inspired co-differentiation, will be required. The goal is not simply to keep organoids alive longer, but to enable them to progress toward authentic late milestones — such as layered cortical plates, neuronal maturation, and functional circuits — that can be studied reproducibly and at scale. Achieving this will elevate brain organoids from mid-gestation models to comprehensive, experimentally tractable systems for exploring human cortical development and its disorders.

*This work was supported by the Pathway to Independence Award from the NINDS (4R00NS135123-02) (to XQ).*
